# Frailty Syndrome and Depressive Symptoms in Patients with Ischemic Heart Disease

**DOI:** 10.3390/diagnostics16111707

**Published:** 2026-06-02

**Authors:** Kristina Krivoshapova, Daria Tsygankova, Anastasia Neeshpapa, Evgeny Bazdyrev, Victoria Karetnikova, Olga Barbarash

**Affiliations:** Research Institute for Complex Issues of Cardiovascular Diseases, Kemerovo 650002, Russia

**Keywords:** frailty syndrome, depressive symptoms, ischemic heart disease, PRISMA-7 questionnaire, GDS-15 geriatric depression scale

## Abstract

**Background/Objectives**: This study aims to evaluate the association between frailty syndrome and depressive symptoms within a cohort of patients with ischemic heart disease (IHD). **Methods**: This single-center cross-sectional study included 169 patients with IHD admitted for elective percutaneous coronary intervention. The median age was 68.00 [63.00–73.00] years; 59.7% of the patients were male. Frailty screening was performed using the European PRISMA-7 questionnaire, where a score of 3 or higher indicated a high probability of frailty. Depressive symptoms were assessed using the 15-item Geriatric Depression Scale (GDS-15). The total GDS-15 score was interpreted as follows: 0–5 points indicated no depressive symptoms, 6–10 points indicated mild depression, and 11–15 points indicated severe depression. **Results**: The prevalence of frailty in the study cohort, based on the PRISMA-7 questionnaire, was 52.7%. The Geriatric Depression Scale (GDS-15) indicated a high probability of depressive symptoms in 19.5% of patients with IHD. The prevalence of depressive symptoms was significantly higher in frail than in non-frail patients. Furthermore, depressive disorders were 2.913 times more frequent among elderly frail patients with IHD compared to non-frail patients (95% CI: 1.262–6.725; *p* = 0.010). Correlation analysis confirmed a direct positive relationship between PRISMA-7 scores and GDS-15 scores (ρ = 0.392; *p* < 0.001). **Conclusions**: A significant association was identified between frailty and depressive symptoms in patients with IHD.

## 1. Introduction

Cardiovascular diseases (CVDs) account for more than 17 million deaths worldwide annually, representing approximately 29.0% of total mortality [1]. The prevalence of CVD increases proportionally with age [2]. Given the steady rise in life expectancy, the coming decades are expected to see a surge not only in CVD incidence but also in the prevalence of frailty syndrome [3,4,5]. Frailty is a progressive geriatric syndrome arising from a decline in the body’s physiological reserve, leading to an increase in a person’s vulnerability to the effects of various exogenous risk factors [6]. Frailty is primarily associated with various adverse health outcomes, including disability, a high risk of CVD, and increased mortality [7]. Frailty has been linked to chronic inflammation, which leads to endothelial dysfunction and increased atherosclerotic risk [8]. Behavioral manifestations of frailty, such as reduced physical activity and social withdrawal, can create a cycle that further amplifies the likelihood of developing CVD [9].

Frailty among middle-aged and elderly populations is frequently associated with depressive disorders [10], which may not only share common risk factors with CVD but also mediate the relationship between frailty and CVD. Depressive symptoms may be linked to increased inflammation, metabolic changes, and autonomic dysfunction [11,12]. Furthermore, depression can lead to behavioral changes, such as physical inactivity, poor dietary habits, and heightened stress responses, which may exacerbate pathological aging and increase CVD risk [9]. Thus, depressive disorders may serve both as a mediator between frailty and CVD and as a modifiable target for intervention to reduce frailty progression among patients with CVD [13]. Given the potential role of depressive symptoms in mediating the link between frailty and CVD, studying the specifics of this relationship is crucial for developing targeted interventions.

The association between frailty and depressive disorders has been evaluated in several cross-sectional and longitudinal studies. Hypotheses regarding comparable biological mechanisms underlying the development of frailty and depression have been proposed [14]. While cross-sectional results indicate a positive association between depression and frailty [15,16], data from cohort studies are less consistent [17]. Furthermore, several studies investigating the bidirectional relationship between depression and frailty have yielded contradictory results [18,19,20]. The prevalence of frailty and depression in patients with ischemic heart disease (IHD) varies widely, ranging from 20% to 60%, depending on the assessment method used [21,22,23,24], which significantly exceeds comparable figures in the general population. Studies examining the relationship between frailty and depression in patients with IHD are extremely scarce, and their results are equally inconsistent, possibly due to differences in the study populations, follow-up duration, frailty assessment tools, and methods for measuring depressive symptoms [21,25,26]. Based on the above, our study aims to further investigate the relationship between frailty and depressive symptoms in patients with IHD.

## 2. Materials and Methods

This single-center cross-sectional study included 169 patients with IHD admitted to the Cardiology Department of the Federal State Budgetary Scientific Institution Research Institute for Complex Issues of Cardiovascular Diseases (Kemerovo) for elective percutaneous coronary intervention (PCI) between 2023 and 2024. All patients were informed about the study and provided written informed consent to participate in the study, which was approved by the local Ethics Committee of the Research Institute for Complex Issues of Cardiovascular Diseases (Kemerovo), Protocol No. 12 dated 27 December 2019. The diagnosis of IHD was verified based on the 2019 European Society of Cardiology (ESC) guidelines for the diagnosis and management of chronic coronary syndromes [27], taking into account anginal chest pain or its equivalent, medical history, and instrumental diagnostic methods, including electrocardiography, echocardiography, ambulatory electrocardiography monitoring, and coronary angiography. Frailty was screened using the European PRISMA-7 questionnaire, where each positive response was assigned 1 point. A total score of 3 or higher indicated a high probability of frailty [28]. Emotional state, interest in life, energy levels, and other symptoms of depression were assessed using the 15-item Geriatric Depression Scale (GDS-15). Total GDS-15 scores were interpreted as follows: 0–5 points, no depressive symptoms; 6–10 points, mild depression; and 11–15 points, severe depression [29].

The sample was formed in accordance with the main inclusion criteria: age exceeding 60 years; stable IHD at the time of hospitalization (angina functional class I–III and/or post-infarction cardiosclerosis); and elective PCI is reported as the reason for hospitalization. The following exclusion criteria were applied: neuromuscular diseases; functional class IV chronic heart failure; uncontrolled arterial hypertension (AH); concomitant IHD and valvular heart disease; presence of severe comorbidities impairing the patient’s mental or somatic status; traumatic brain injuries; inability to understand and (or) follow the study protocol procedures; and refusal (or withdrawal of consent) to participate in the study.

To determine the required sample size, an a priori power calculation was performed. Using the parameters of *p* = 0.05, power = 0.80, and d = 0.5, the required sample size for each group was 64 participants, yielding a total sample size of 128 participants. Statistical analysis of the study results was performed using the IBM SPSS Statistics 26.0.0 (USA) software package. Qualitative variables are described using absolute and relative values (*n*, %). The normality of the distribution of quantitative variables was assessed using the Shapiro–Wilk test. Most quantitative variables did not follow a normal distribution (Shapiro–Wilk W 0.7–0.9 criteria; *p* < 0.05) and are presented as median and interquartile range (Me [Q25–Q75]). Quantitative variables with a normal sampling distribution were described using arithmetic means (M) and standard deviations (SDs). The boundaries of the 95% confidence interval (CI) were specified as a measure of representativeness for the mean values. Pearson’s chi-squared test was used to assess the statistical significance of differences in qualitative variables between two independent groups. In cases where the number of expected observations in any cell of a 2 × 2 contingency table was less than 5, Fisher’s exact test was used to evaluate the significance level of the differences. The Mann–Whitney U test was applied to compare two independent groups for quantitative variables. A predictive model was constructed using binary logistic regression with the backward stepwise variable elimination method (Wald test). ROC analysis was used to evaluate the diagnostic performance of the predictive model and to determine the optimal classification threshold value. The quality of the predictive model was assessed based on the area under the ROC curve (AUC) values with standard error, 95% CI, and statistical significance level. The direction and strength of the correlation between two quantitative variables were evaluated using the Spearman rank correlation coefficient (for non-normal distributions). A predictive model characterizing the dependence of a quantitative variable on factors was developed using the linear regression method. Differences were considered statistically significant at *p* ≤ 0.05.

## 3. Results

The median age in the study cohort was 68.0 years [63.0–73.0], and 59.7% of the patients were male. Almost all patients (161 (95.3%) individuals) had a history of AH, and half had a history of myocardial infarction (85 (50.3%) patients). Atrial fibrillation (AF) was identified in 29 (17.1%) patients. Type 2 diabetes mellitus (T2DM) was found in approximately one-third of the patients (50 (29.6%) individuals), and the majority were overweight or obese, with a body mass index (BMI) of 29.43 ± 5.81 kg/m^2^. A history of stroke (acute cerebrovascular accident (ACVA)) was present in 16 (9.5%) patients. According to echocardiography data, the median left ventricular ejection fraction was 58.0% [41.50–64.50%]. Coronary angiography revealed double- or triple-vessel coronary artery disease in the majority of patients; meanwhile, half of the study cohort had previously undergone a revascularization procedure. The prevalence of frailty according to the PRISMA-7 questionnaire was 52.7%, with a median score of 3.00 [2.00–3.00]. In 19.5% of cases, the Geriatric Depression Scale (GDS-15) indicated a high probability of depressive symptoms among patients with IHD prior to elective PCI, with a median score of 3.00 [2.00–5.00] (Table 1).

A comparative analysis of the groups of patients with and without frailty showed that prior to elective PCI, older women were significantly more likely to be frail. In the patients’ medical history, myocardial infarction, T2DM, AF, stroke, COPD, and prior PCI occurred with equal frequency. Results of laboratory diagnostic tests showed lower blood hemoglobin levels in frail patients with IHD compared to patients without frailty (130.11 ± 12.79 (127.42–132.81) g/L and 135.22 ± 14.32 (132.04–138.41) g/L, respectively; *p* = 0.015, Table 2). At the same time, manifestations of depressive symptoms were more characteristic of frail patients with IHD; specifically, mild depression was identified in 8 (10.0%) patients without frailty and 21 (23.6%) patients with frailty, while severe depression was found in 1 (1.2%) patient without frailty and 3 (3.4%) patients with frailty. The odds of having depression were 2.913 times higher among frail patients with IHD compared to patients without frailty; the odds difference was statistically significant (95% CI: 1.262–6.725, *p* = 0.010). No other statistically significant differences were found.

To identify associations between frailty in patients with IHD and quantitative and qualitative predictors studied, a binary logistic regression method with stepwise variable elimination (Wald test) was used. As a result of the analysis, a predictive model was developed to determine the probability of frailty according to the PRISMA-7 questionnaire, depending on the age of the patients in the study sample and depressive disorders according to the GDS-15 geriatric depression scale; the observed relationship is described by Equation (1):

p = 1/(1 + e^−z^) × 100%, (1)

z = −4.960 + 0.072X_Age_ + 1.208X_Depression_,

Here, p is the presence of frailty according to the PRISMA-7 questionnaire; z is the value of the logistic function; X_Age_ is the age of the patients with IHD (years); and X_Depression_ is the presence of depressive disorders according to the GDS-15 geriatric depression scale.

The resulting regression model is statistically significant (*p* < 0.001). Nagelkerke’s pseudo-R^2^ was 14.5%.

With each 1-year increase in the age of patients with IHD, the odds of detecting frailty according to the PRISMA-7 questionnaire increased by 1.074 times. Upon detection of depressive disorders according to the GDS-15 geriatric depression scale, the odds of the presence of frailty increased by 3.346 times (Table 3).

**Figure 1:**
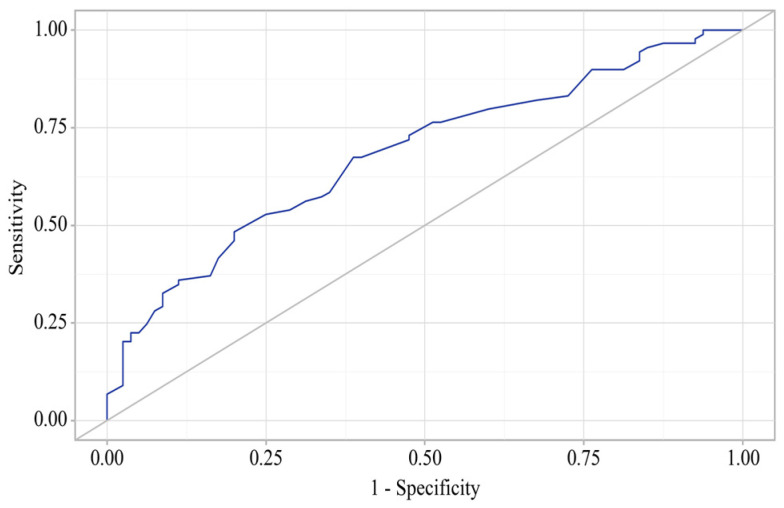
ROC curve characterizing the discriminatory power of the regression model in predicting frailty syndrome according to the PRISMA-7 questionnaire.

The curve obtained when assessing the discriminatory power of the regression model using ROC analysis is illustrated in Figure 1.

The probability *p* is a statistically significant predictor of frailty based on the PRISMA-7 questionnaire (AUC = 0.682; 95% CI: 0.602–0.761, *p* < 0.001). The threshold value for the probability *p* at the cut-off point, which corresponded to the highest Youden’s index value, was 0.499. The sensitivity and specificity of the resulting predictive model were 67.4% and 61.3%, respectively.

**Figure 2:**
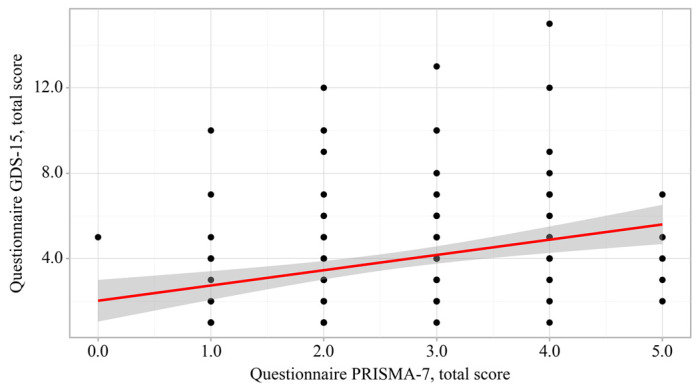
Graph of the regression function characterizing the dependence.

Next, a correlation analysis was performed to examine the relationship between scores from the PRISMA-7 questionnaire and the GDS-15 geriatric depression scale. The Spearman rank correlation coefficient was applied to assess both the direction and strength of the association linking these two quantitative measures. To further explore the dependence of the quantitative variable on relevant factors, a predictive model was developed using linear regression analysis. The correlation analysis established a direct relationship of moderate strength (Table 4, Figure 2).

The observed relationship is described by the simple linear regression in Equation (2):

Y_GDS-15, total score_ = 0.715 × X_PRISMA-7_, total score + 2.023, (2)

With an increase of 1 in the total score on the PRISMA-7 questionnaire, an increase of 0.715 in the total score on the GDS-15 geriatric depression scale should be expected. The resulting model explains 13.0% of the observed variance.

Thus, the final logistic regression model included only patient age and the presence of depressive symptoms according to the GDS-15 geriatric depression scale as predictors of frailty among all the studied quantitative and qualitative variables. The resulting regression model showed moderate discriminatory ability (Nagelkerke’s pseudo-R^2^ = 14.5% and AUC = 0.682). Correlation analysis confirmed a moderate direct relationship between PRISMA-7 scores and GDS-15 scores (ρ = 0.392; *p* < 0.001). These results indicate associations between depressive symptoms and frailty in patients with IHD, but further research is required in this area, given the small sample size and cross-sectional nature of the study. It should also be noted that the PRISMA-7 screening questionnaire and the GDS-15 geriatric depression scale are two different tools for assessing patient status. However, they share several common features, particularly in areas such as decreased physical activity and the detection of severe fatigue. This may lead to an overestimation of the relationship between the factors being studied. However, there are currently no universal approaches to diagnosing frailty and depression.

## 4. Discussion

Based on the results of our study and previously conducted research, it can be concluded that depression is one of the most important factors associated with frailty syndrome. However, the pathophysiological mechanisms underlying the significant associations between frailty and depression in patients of various age categories remain unknown. Chronic inflammation, oxidative stress, and mitochondrial dysfunction may serve as common biological pathways for the development of frailty and depression [30,31]. An unhealthy lifestyle—including smoking, excessive alcohol consumption, unbalanced nutrition, and low physical activity—may contribute to the bidirectional link between these pathological conditions [32].

In this study, significant associations were identified between frailty (as measured by the PRISMA-7 questionnaire) and the presence of depressive symptoms (according to the GDS-15 geriatric depression scale) in patients with IHD (odds ratio (OR), 3.3 (95% CI: 1.3–8.0)). The obtained results are consistent with a number of previously conducted works. A five-year study of 167,729 individuals in the Netherlands found that depression and anxiety were significantly associated with frailty, assessed via the deficit accumulation model (OR, 1.7 (95% CI: 1.3–2.3)) [33]. Zhu J. and colleagues [34], as a result of their study, concluded that reduced grip strength (OR, 1.23; *p* = 0.003), slower walking pace (OR, 1.55; *p* = 0.027), and low levels of physical activity (OR, 1.44; *p* = 0.003) contribute to the development of depression. Since all these factors are potentially modifiable, increasing muscle strength and physical activity may be effective methods for preventing and treating both depressive symptoms and frailty. Using the frailty phenotype, a multiple linear regression analysis in 1789 elderly Chinese participants demonstrated a significant independent association between depressive symptoms and frailty (β = 0.272, *p* < 0.001), consistent with our earlier results [35].

Maştaleru A. and colleagues [36] conducted a retrospective study to investigate the relationship between symptoms of depression and frailty in an elderly population, which showed that the prevalence of depression, using the GDS-15 scale, was 66.7%, which is slightly higher than the results of our study and those reported in previously conducted studies; furthermore, a significant correlation was observed between depression and frailty screened using the Fried L.P. phenotypic model. In the work by Borges M.K. and colleagues [37], associations were discovered between frailty and symptoms of depression among outpatient geriatric patients, regardless of the diagnostic tools used. Ribeiro O. and colleagues [38] used data from two Portuguese studies among long-livers and found that depression is significantly more common in pre-frail and frail long-livers. In another prospective cohort study among a population over 60 years of age, significant associations were also found between frailty syndrome and symptoms of depression [10].

When conducting research in this field, several questions arise regarding whether frailty syndrome or depression is the initiating agent and what common mechanisms might underlie the development of these pathological conditions. In our study, correlation analysis confirmed a direct relationship between the severity of frailty manifestations and depression. A cross-sectional study involving 5844 individuals from seven cities in China found that depression was significantly associated with the development of frailty [39]. Results from studies among the elderly populations of Brazil and Latin America also showed that depression increases the risk of detecting frailty syndrome by 2–3 times, as was found in our study [40,41]. These findings contradict the results of a longitudinal study [42]. In a cross-sectional study of 576 Chinese individuals aged 65 years and older, frailty was significantly associated with depressive symptoms [43]. The inconsistency of the obtained results may be due to different methods used for diagnosing frailty and depressive disorders. Furthermore, a study investigating longevity and aging (RuLAS), conducted among 1168 Chinese individuals aged 70 and older, found that depressive symptoms were significantly associated with the development of frailty syndrome (OR, 2.79 (95% CI: 1.09–7.10)) [44].

There are very few studies in this area among patients with IHD, and the results of our study certainly add to this limited data. Huang J. and colleagues [45] found that frailty was a factor associated with depressive symptoms in elderly heart failure patients. A systematic review of studies examining the associations between frailty and depression among elderly patients with IHD yielded a pooled OR of 2.45 (95% CI: 1.22–4.92) [46], which is consistent with the results of our study. Moreover, in a similar meta-analysis among the general elderly population, the OR was significantly lower—1.62 (95% CI: 1.28–2.04) [26]. A possible reason for the more frequent occurrence of depressive symptoms and frailty in patients with IHD may be shared biological mechanisms, primarily chronic inflammation. Elevated levels of proinflammatory cytokines may promote atherosclerosis, accelerate muscle catabolism, lead to frailty, and disrupt dopaminergic neurotransmission, which underlies depressive disorders [47,48]. Mitochondrial dysfunction and oxidative stress cause energy metabolism imbalances, which jointly contribute to physical decline and mood disorders [49]. Complex pathophysiological interactions may create a potential vicious cycle that significantly worsens the prognosis of patients with IHD compared with the general elderly population [25,50]. Given the high incidence of comorbidity of depression and frailty in patients with IHD, and the elevated risk of adverse outcomes associated with their synergistic effect, further research is needed to identify potential mechanisms underlying their relationship.

## 5. Conclusions

The results of the conducted study confirm the presence of significant associations between frailty and symptoms of depression in elderly patients with IHD. The present findings support the routine screening for frailty and depressive symptoms in clinical practice. However, longitudinal studies are required to determine whether this approach, combined with targeted interventions, leads to improvements in clinical outcomes.

This study has several limitations. These include, in particular, the small sample size, the cross-sectional nature of the study, and the use of only one tool for screening frailty (the PRISMA-7 questionnaire) and depressive symptoms (the GDS-15 scale). These limitations, taken together, may have potentially contributed to the confounding effects of these factors.

## Figures and Tables

**Table 1:** Clinical and laboratory characteristics of patients with ischemic heart disease.

Parameters	Patient Characteristics (*n* = 169)
Median age, years, Me [Q1–Q3]	68.00 [63.00–73.00]
Men, *n* (%)	101 (59.7)
BMI, kg/m^2^, M ± SD	29.43 ± 5.81
Smoker/quit less than 3 months ago, *n* (%)	46 (27.2)
AH, *n* (%)	161 (95.3)
CHF II–III, *n* (%)	151 (89.3)
LVEF, %, Me [Q1–Q3]	58.00 [41.50–64.50]
PICS, *n* (%)	85 (50.3)
History of ACVA, *n* (%)	16 (9.5)
AF in the preoperative period, *n* (%)	29 (17.1)
T2DM, *n* (%)	50 (29.6)
History of PCI, *n* (%)	82 (48.5)
1-vessel coronary artery disease, *n* (%)	38 (22.5)
2-vessel coronary artery disease, *n* (%)	60 (35.5)
3-vessel coronary artery disease, *n* (%)	71 (42.0)
COPD, *n* (%)	7 (4.1)
Depressive symptoms, *n* (%)	33 (19.5)
Geriatric Depression Scale, GDS-15, total score, Me [Q1–Q3]	3.00 [2.00–5.00]
Frailty, *n* (%)	89 (52.7)
PRISMA-7 questionnaire, total score, Me [Q1–Q3]	3.00 [2.00–3.00]
Hemoglobin, g/L, M ± SD	132.53 ± 13.73
Glucose, mmol/L, Me [Q1–Q3]	5.70 [5.20–6.55]
Creatinine, µmol/L, Me [Q1–Q3]	89.00 [77.00–101.00]
Total cholesterol, mmol/L, Me [Q1–Q3]	4.00 [3.42–4.97]

Note: BMI, body mass index; AH, arterial hypertension; CHF, chronic heart failure; LVEF, left ventricular ejection fraction; PICS, post-infarction cardiosclerosis; ACVA, acute cerebrovascular accident; AF, atrial fibrillation; T2DM, type 2 diabetes mellitus; PCI, percutaneous coronary intervention; COPD, chronic obstructive pulmonary disease.

**Table 2:** Clinical and laboratory characteristics of frail patients with stable ischemic heart disease.

Parameter	Group of Patients Without Frailty, *n*_0_ = 80 (47.3%)	Group of Patients with Frailty, *n*_1_ = 89 (52.7%)	*p*
Median age, years, Me [Q1–Q3]	66.00 [62.00–71.00]	69.00 [64.00–75.00]	0.002 *
Men, *n* (%)	57 (71.2)	44 (49.4)	0.004 *
BMI, kg/m^2^, M ± SD (95% CI)	28.45 ± 4.89 (27.36–29.54)	30.31 ± 6.42 (28.96–31.66)	0.035 *
Smoker/quit less than 3 months ago, *n* (%)	22 (27.5)	24 (27.0)	0.938
AH, *n* (%)	77 (96.2)	84 (94.4)	0.723
CHF II–III, *n* (%)	66 (82.5)	85 (95.5)	0.058
LVEF, %, Me [Q1–Q3]	59.50 [47.25–64.25]	54.00 [41.00–64.00]	0.339
PICS, *n* (%)	41 (51.2)	44 (49.4)	0.814
History of ACVA, *n* (%)	5 (6.2)	11 (12.4)	0.199
AF in the preoperative period, *n* (%)	12 (15.0)	17 (19.1)	0.480
T2DM, *n* (%)	20 (25.0)	30 (33.7)	0.216
History of PCI, *n* (%)	40 (50.0)	42 (47.2)	0.715
1-vessel coronary artery disease, *n* (%)	18 (22.5)	20 (22.5)	0.702
2-vessel coronary artery disease, *n* (%)	26 (32.5)	34 (38.2)	
3-vessel coronary artery disease, *n* (%)	36 (45.0)	35 (39.3)	
COPD, *n* (%)	4 (5.0)	3 (3.4)	0.709
Depressive symptoms, *n* (%)	9 (11.2)	24 (26.9)	0.036 *
Geriatric Depression Scale, GDS-15, total score, Me [Q1–Q3]	2.00 [1.00–4.00]	4.00 [3.00–6.00]	<0.001 *
PRISMA-7 questionnaire, total score, Me [Q1–Q3]	2.00 [1.00–2.00]	3.00 [3.00–4.00]	<0.001 *
Hemoglobin, g/L, M ± SD	135.22 ± 14.32 (132.04–138.41)	130.11 ± 12.79 (127.42–132.81)	0.015 *
Glucose, mmol/L, Me [Q1–Q3]	5.60 [5.20–6.40]	5.80 [5.20–6.60]	0.472
Creatinine, µmol/L, Me [Q1–Q3]	89.50 [76.75–100.25]	88.00 [78.50–101.00]	0.791
Total cholesterol, mmol/L, Me [Q1–Q3]	3.90 [3.50–4.80]	4.30 [3.45–5.00]	0.410

Note: *, statistically significant differences, *p* ≤ 0.05.

**Table 3:** Characteristics of the association between model predictors and the odds of identifying frailty syndrome according to the PRISMA-7 questionnaire.

Predictors	Unadjusted		Adjusted	
	COR; 95% CI	*p*	AOR; 95% CI	*p*
Age, years	1.069; 1.026–1.113	0.001 *	1.074; 1.030–1.121	0.001 *
Presence of depressive symptoms according to the GDS-15 scale	2.913; 1.261–6.726	0.012 *	3.346; 1.385–8.085	0.007 *

Note: *, predictor influence is statistically significant, *p* ≤ 0.05.

**Table 4:** Results of the correlation analysis of the relationship between scores on the PRISMA-7 questionnaire and the GDS-15 geriatric depression scale.

Parameter	Correlation Characteristic		
	ρ	Strength of Association (Chaddock Scale)	*p*
PRISMA-7, total score— GDS-15, total score	0.392	Moderate	<0.001 *

Note: *, statistically significant differences, *p* ≤ 0.05.

## Data Availability

The original contributions presented in this study are included in the article. Further inquiries can be directed to the corresponding author.

## Abbreviations

ACVA	Acute cerebrovascular accident
AH	Arterial hypertension
AF	Atrial fibrillation
BMI	Body mass index
CVD	Cardiovascular disease
CHF	Chronic heart failure
CI	Confidence interval
COPD	Chronic obstructive pulmonary disease
IHD	Ischemic heart disease
LVEF	Left ventricular ejection fraction
OR	Odds ratio
PCI	Percutaneous coronary intervention
PICS	Postinfarction cardiosclerosis
T2DM	Type 2 diabetes mellitus
